# Characterization of the complete mitochondrial genome of *Coprinellus micaceus*, a wild saprobic mushroom in China

**DOI:** 10.1080/23802359.2021.1938717

**Published:** 2021-06-14

**Authors:** Shu-rong Wang, Jiang-ping Zhang, Yi-rong He, Ming-chang Chang, Jun-long Meng

**Affiliations:** a College of Food Science and Engineering, Shanxi Agricultural University, Taigu, PR China; b Shanxi Forestry Vocational and Technical College, Taiyuan, PR China

**Keywords:** Fungi, *Coprinellus micaceus*, mitogenome, evolution

## Abstract

The Glistening Inkcap (*Coprinellus micaceus*) is a wild saprobic mushroom in China. In this study, we assembled and annotated its complete mitochondrial genome using raw data sequenced through Illumina NovaSeq 6000 platform (Illumina, San Diego, CA). The length of the *C*. *micaceus* mitochondrial genome is 65,450 bp with 33.05% GC content. Totally, 41 genes, including 14 protein-coding genes, 25 tRNAs and 2 rRNAs were identified in the mitochondrial genome. Phylogenetic analysis showed that the mitochondrial genome relationship between *C. micaceus* and *Coprinopsis cinerea* was the closest.

Glistening Inkcap mushroom [*Coprinellus micaceus* (Bull.) Vilgalys, Hopple & Jacq. Johnson 2001], formerly named as Shaggy Inkcap [*Coprinus micaceus* (Bull.) Fr. 1838], is a common fungal species belonging to Psathyrellaceaea family (Kirk et al. 2008). The sporophore of the saprobe typically grow in clusters on or near rotting hardwood tree stumps or roots. This edible mushroom is potentially poisonous if collected from roadsides or polluted land for the reason that its mycellium can assimilate and accumulate high levels of heavy metals such as Ca, Co, and Fe (Sarikurkcu et al. 2020). The microscopic characteristics and cytogenetics of *C. micaceus* are well studied, and it has been used frequently as a model organism to study cell division and meiosis in Basidiomycetes (Thielke 1982). Many antimicrobial and enzyme-inhibiting compounds have been isolated from its sporophore (Zahid et al. 2006; Ayodele and Idoko 2011; Nguyen et al. 2014), however, no complete mitochondrial genome (mitogenome) is available to date for the species. In the present study, to provide genetic information of *C*. *micaceus*, we assembled and annotated its complete mitogenome, and performed a phylogenetic analysis of related taxa of this species.

The specimen of *Coprinellus micaceus* was isolated from the plant garden of Shanxi Agricultural University (Taigu County, Shanxi Province, PR China, 37°42'1.757″N; 112°57'7.359″E) and a strain was deposited in the Herbarium of Mycology of Shanxi Agricultural University (HMSAU, Wang Shu-rong, wangsr2015@126.com) under the voucher number HMSAU20073. The mycelium genomic DNA of the Glistening Inkcap mushroom was extracted by using CTAB method and stored at the Herbarium of Mycology of Shanxi Agricultural University (HMSAU, No. HMSAU-W01). Whole genome shotgun sequencing of 350 bp DNA library was performed on the Illumina NovaSeq 6000 platform by Wuhan Benagen Tech Solutions Co., Ltd., Wuhan, China to generate approximately 1.7 Gb of raw data of 150-bp-long paired-end read. A total of 29,747,624 reads were subjected to quality control and trimming using SOAPnuke 1.3.0 (Chen et al. 2018), which removed reads containing 50% low-quality bases (quality value, ≤5) and overlaps with adapter sequence, generating a total of 29,702,202 clean reads. The assembly was completed with SPAdes (version: 3.14.0; parameter: -k 127) (Bankevich et al. 2012) by using the mitogenome of *Coprinopsis cinerea* (NW 003307477.1, Stajich et al. 2010) as the initial reference. The annotation was performed with MAKER 3.01.03 (Cantarel et al. 2007).

The complete mitogenome of *Coprinellus micaceus* was 64,450 bp in length with a GC content of 33.05% and the depth of Illumina sequence averaged 275.57×. The annotated mitogenome was submitted to Genbank under accession number MW291577. In total, 41 genes were annotated, including 14 protein coding genes, 25 tRNA genes, and 2 rRNA genes (*rrnS* and *rrnL*). The 14 conserved protein coding genes respectively encoded the seven ubiquinone reductase subunits of NADH (*nad1*, *nad2*, *nad3*, *nad4*, *nad4L*, *nad5*, and *nad6*), three cytochrome oxidase subunits (*cox1*, *cox2*, and *cox3*), three ATP synthase subunits (*atp6*, *atp8*, and *atp9*) and the apocytochrome b (cob). Sequences of 14 protein coding genes were separately aligned using Muscle algorithms in MEGA X (Kumar et al. 2018). Evolutionary models were compared using MEGA X, and since the LG + G + F model received the best ln(L) score in all cases, this model was used in maximum-likelihood (ML) analyses implemented in MEGA X for the combined dataset. Result showed the relationship between *Coprinopsis* and related genus in Agaricales (Figure 1) agrees well with previous studies based on a multilocus phylogenetic overview (Matheny et al. 2006). It also showed that the relationship between the *Coprinopsis cinerea* (NW003307477.1, Stajich et al. 2010) and *C*. *micaceus* mitogenome is the closest, which is consistent to the previous study based on rDNA ITS barcode sequences for Psathyrellaceae including *Coprinellus* and *Coprinopsis*. (Meghna et al. 2018). The size of the complete mitogenome (64,450 bp) of *C. micaceus* was larger than that of *Coprinopsis_cinerea*, 42,448 bp (NW003307477.1, Stajich et al. 2010). Recent researches indicated that the genome sizes of mitochondria in Agaricales varied significantly from about 40 kb (*Paxillus involutus*, MK993563, Li et al. 2020) to more than 120 kb (*Clavaria fumosa*, MT114157, Wang et al. 2020), sometimes that were near in a same genera or very closed genus (Yang et al. 2016；Li et al. 2018, 2019), but not for others (Huang et al. 2021). This study will help to better understand the phylogenetic status of *Coprinellus* and the mitogenome evolution in macrofungi, and will be of interest for further applied researches by selecting the most suitable species for biotechnological and nutritional interest.

**Figure 1.**
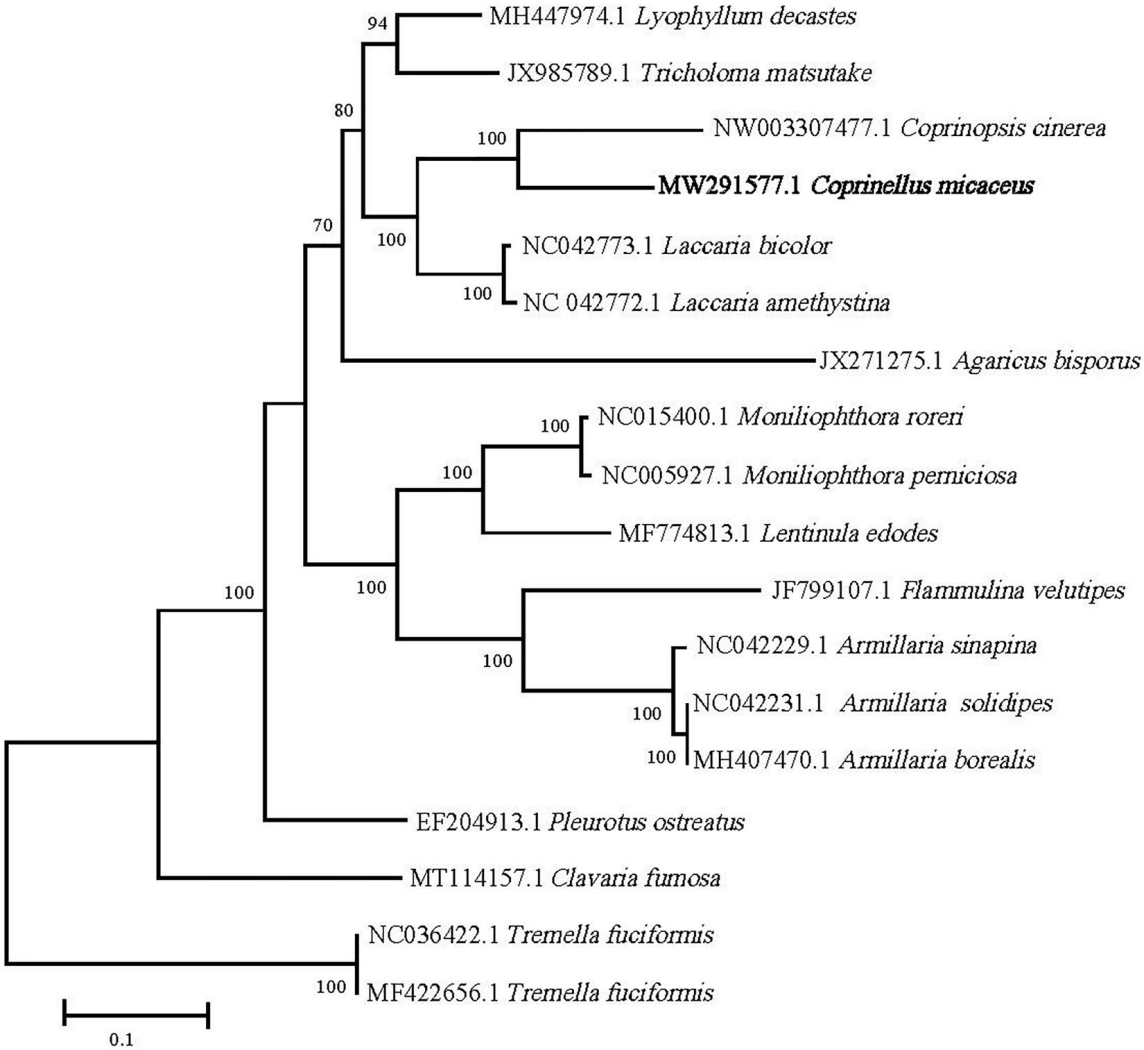
ML phylogenetic tree constructed based on 14 PCGs of *C. micaceus* and 15 fungi species from the Agaricales with *Tremella fuciformis* from the Tremellales as outgroup. The phylogenetic tree was constructed by using MEGA X (bootstrap values based on 100 replications). Numbers near the nodes are bootstrap values.

## Data Availability

The genome sequence data that support the findings of this study are openly available in GenBank of NCBI at https://www.ncbi.nlm.nih.gov under the Accession no. MW291577. The associated BioProject, SRA, and Bio-Sample numbers are PRJNA682905, SRP297495, and SAMN17013754, respectively.
